# Impact of Neutrophil Extracellular Traps on Clinical Outcome after Subarachnoid Hemorrhage: A Translational Narrative Review

**DOI:** 10.1007/s12975-026-01461-6

**Published:** 2026-06-30

**Authors:** François Delvoye, Julie Lebeau, Cécile Oury, Céline D’Emal, Bernard Lambermont, Nathalie Layios, Didier Ledoux, Annie Dubuisson, Louis Deprez, Martin Moïse, Benoit Ho-Tin-Noé, Mikael Mazighi, Nicolas Engrand, Jean-Philippe Désilles, Benjamin Maïer

**Affiliations:** 1 Interventional Neuroradiology Department, Rothschild Foundation Hospital, Paris, France; 2 Neurology Department, CHU Sart-Tilman, CHU of Liège, 1 Avenue de l’Hôpital, Liège, 4000 Belgium; 3 Laboratory of Cardiology, GIGA Research Institute, University of Liège, Liège, Belgium; 4 Neurosurgery Department, CHU Sart-Tilman, Liège, Belgium; 5 Neuro-intensive Care Unit, CHU Sart-Tilman, Liège, Belgium; 6 Interventional Neuroradiology Department, CHU Sart-Tilman, Liège, Belgium; 7 Therapeutic Optimization in Neuropsychopharmacology, Université Paris-Cité, INSERM, Paris, U1144 France; 8 Neuro-intensive Care Unit, Rothschild Foundation Hospital, Paris, France; 9 Stroke-Link, F-CRIN Research Infrastructure, Lille, France; 10 Neurology Department, Hôpital Paris Saint-Joseph, Paris, France

**Keywords:** Neutrophil Extracellular Traps, Subarachnoid Hemorrhage, Thrombo-Inflammation, Delayed Cerebral Ischemia, Cognitive Impairment

## Abstract

Aneurysmal subarachnoid hemorrhage (aSAH) remains associated with substantial morbidity and mortality despite best medical treatment. Increasing evidence implicates thrombo-inflammation (TI), driven by neutrophils and neutrophil extracellular traps (NETs), in the pathogenesis of delayed cerebral ischemia (DCI) and cognitive impairment. This narrative review summarizes experimental and clinical data linking neutrophil activation and NETs formation to DCI after aSAH and outlines emerging therapeutic strategies. This review was structured according to the SANRA (Scale for the Assessment of Narrative Review Articles) guidelines and followed general recommendations for narrative reviews. A comprehensive PubMed and clinicaltrials.gov search identified experimental and clinical studies published in English without time window, focusing on TI, neutrophil activation, or NETosis in the acute phase of aSAH. Past and ongoing clinical trials were classified by mechanism of action. Experimental models and human data show early and sustained neutrophil activation within cerebral and systemic compartments after aSAH. NETs promote microvascular thrombosis, blood-brain barrier disruption, microglial activation, and neuronal injury, ultimately contributing to DCI and long-term cognitive impairment. Outside the brain, NETs may also aggravate neurogenic pulmonary edema and cardiac dysfunction. In animal models, deoxyribonuclease I (DNase I) effectively degrades NETs, improving perfusion, limiting neuronal apoptosis, and enhancing recovery. Several ongoing trials target TI pathways, including the RESET trial, which evaluates the impact of IV DNase I on clinical outcome after aSAH. NETs produced after neutrophil activation may be central to DCI. Targeting NETs represents a promising translational path to improve cerebral and systemic outcomes after aSAH.

**Supplementary Information:**

The online version contains supplementary material available at https://doi.org/10.1007/s12975-026-01461-6.

## Introduction

Subarachnoid hemorrhage due to aneurysm rupture (aSAH) is associated with substantial morbidity and mortality. Mortality is estimated at 14% [1], while 19% of survivors [2] will not regain full autonomy, and 67% will report reduced quality of life [3]. More importantly, the proportion of patients unable to resume their usual activities after SAH ranges from 40% to 82% across series [4].

Many patients also experience a measurable decline in quality of life [5] and a substantial proportion, despite returning to work and maintaining apparent functional independence, requires specific accommodations [4].

Given these individual consequences, the broader societal impact is considerable: the disease affects people during their most productive years [6] and generates substantial economic losses, partly driven by persistent attentional deficits [7].

A substantial part of this morbidity and mortality (including fatigue [8]) may relate to thrombo-inflammation (TI), both cerebral [9] and peripheral [10] (i.e. pulmonary), arising in the acute and subacute phases of aSAH.

The best-known subacute cerebral and clinically noticeable phenomenon related to TI is delayed cerebral ischemia (DCI) [11], although as discussed below, low-grade and subclinical TI is also likely responsible for reported morbidity, notably cognitive [12]. DCI is defined as the appearance, between 4 and 21 days after aSAH, of a focal neurological deficit or a decrease in Glasgow Coma Scale (GCS; at least 2 points for at least 1 h) that does not occur immediately after aneurysm exclusion and is not attributable to another cause (ruled out by clinical, laboratory, and radiological means) [13]. The incidence of DCI is estimated to be around 30% [14] − 37% [15], and is significantly associated with an unfavorable 3-month functional outcome [16] and with cognitive impairment [17]. Currently, the only proven preventive treatments for post-aSAH DCI aim to prevent vasospasm or its consequences, for example via nimodipine administration [18] but with overall limited efficacy. Furthermore, based on current evidences, vasospasm is not the sole effector of DCI: up to 21% of patients without vasospasm still develop DCI [19], while in 27% of patients, DCI occurs outside the vascular territory affected by vasospasm [19]. Equally compelling is the reverse observation: 57% of patients with moderate-to-severe vasospasm show no neurological worsening, calling into question a simple causal relationship [20]. TI -and neutrophils in particular- are therefore increasingly regarded as an additional mechanism contributing to DCI, with or without the involvement of concomitant vasospasm, by inducing microcirculatory impairment [21]. Among substances secreted by activated neutrophils, neutrophil extracellular traps, or NETs - a network of decondensed chromatin secreted with histones and pro-inflammatory enzymes - have recently been associated with DCI and clinical prognosis after aSAH [21].

With this as a background, the primary objective of this study was to provide a narrative review of experimental and clinical data regarding the contribution of neutrophils and NETs to TI and DCI following aSAH.

## Methods

### Objective

This review was structured and reported in accordance with the SANRA (Scale for the Assessment of Narrative Review Articles) guidelines [22] and followed general methodological recommendations for narrative reviews [23].

### Search Strategy

The literature search was conducted in two complementary phases to ensure a comprehensive and up-to-date overview of the field.

In the first phase, a systematic search of PubMed was performed to identify relevant pre-clinical and clinical studies published in English without limit on publication date, focusing on TI mechanisms and neutrophil-related pathways in the subacute phase of aSAH, within the time window of DCI that is, up to day 21. The general search strategy as well as the inclusion/exclusion criteria and quality assessment methods are provided in Supplemental Data. A Really Simple Syndication (RSS) feed was also systematically monitored during the preparation phase to capture and integrate the most recent publications relevant to the topic.

In the second phase, we searched clinicaltrials.gov for phase 1 to 3 randomized clinical trials (RCTs) involving pharmacological treatments in the acute phase of aSAH whose mechanism of action targeted TI, at least in part. This complementary approach aimed to map both the published literature and ongoing clinical efforts in the field.

### Study Selection and Data Extraction

Two independent reviewers (FD, JL) screened all titles and abstracts for relevance. We included RCTs, observational studies, preclinical and clinical studies, meta-analyses, and consensus statements addressing the role of neutrophils or NETs in the context of aSAH or related TI processes.

Only studies focusing on the acute or subacute phases of aSAH and investigating pharmacological or mechanistic aspects of neutrophil activation or NETs formation were retained. A few references have been added for clarification and educational purposes.

### Classification of Trials

Completed RCTs were classified as either prematurely terminated (with reasons for discontinuation specified) or carried through to completion. In the latter case, they were further subdivided according to whether they demonstrated success, failure, or absence of published results. Ongoing trials were also identified and classified according to their presumed mode of action and targeted pathway, with their objectives and planned inclusion periods reported when available. The flowchart displaying all RCTs sorted by potential mechanism of action has been added as Supplemental Figs. 2 to 6. In case of multiple targets, the most significant TI pathway has been arbitrarily chosen by authors.

### Methodological Considerations

As this work follows a narrative review design, it does not include a formal risk of bias assessment or quantitative synthesis. While this format allows for broader integration of experimental and clinical evidence, it may be subject to selection bias, which was minimized through comprehensive database searches and inclusion of both published and ongoing studies. This review is complementary to the article recently published by Wroe and colleagues [24], which focused more on biochemicals and intracellular pathways than on clinical data and that we have read with the greatest interest.

## Results

### Parenchymal Injury, its Relationship with Microcirculatory Impairment and Thrombo-inflammation

Recent reviews highlight a significant role of microvascular TI in the pathophysiology of DCI [25, 26]. In animal models, microvascular alterations have been documented immediately after aSAH [25] and are responsible for subsequent ischemia in downstream microvascular territories [27]. Microvascular injury leading to parenchymal lesions probably begins as soon as aSAH occurs [28, 29], triggered by transient global cerebral ischemia (tGCI) [30] during an acute process termed early brain injury (EBI), which is now thought to set the stage for subsequent DCI through activation of TI [31, 32].

In humans, microstructural parenchymal injury, including neuronal damage, has been demonstrated in autopsy series [33, 34], where it is spatially associated with microvascular occlusion. From a temporal perspective, a second peak of microcirculatory injury appears to occur during the DCI time window (7–14 days post-aSAH) [35].

Outside post-mortem studies, only two imaging approaches currently allow a highly imperfect in vivo assessment of microcirculatory and cellular alterations in humans. First, perfusion-based techniques available in digital subtraction angiography (DSA) provide an indirect evaluation of microcirculatory impairment through prolongation of the Time to Peak (TTP). Notably, TTP prolongation at day 9 has recently been identified as a prognostic factor for DCI, independent of the occurrence of severe proximal vasospasm [36]. Second, brain magnetic resonance imaging (MRI) allows the characterization of chronic parenchymal injury and its spatial distribution. In a study performed by Galea and colleagues, MRI at 6 months demonstrated a persistent cortical permeation by hemoglobin, which was correlated with cognitive impairment [37]. Microstructural abnormalities revealed by lower mean kurtosis -the average deviation of water diffusion from a Gaussian distribution- in prespecified gray and white matter areas, are also still detectable on brain MRI 6 weeks after aSAH in patients with cognitive deficits [9]. In contrast, no persistent inflammation could be measured in the same regions at that time, supporting the hypothesis that an early inflammatory process leads to long-lasting microstructural damage [9].

Nonetheless, due to the lack of direct observation of TI phenomenon in humans, we must rely on pre-clinical, predominantly animal, models to elucidate the mechanisms linking microcirculatory dysfunction and parenchymal injury after aSAH.

In murine experimental models of aSAH, microvascular occlusion is persistent and spatially associated with neuronal cell loss [38]. This neuronal injury is particularly pronounced in the CA1 and CA3 regions of the hippocampus, the cerebral cortex, and cerebellar Purkinje cells [39], with maximal damage observed at day 14 [40], coinciding with the second peak of microcirculatory impairment [35] and the highest incidence of DCI.

At the cellular level, neuronal injury results from several complementary mechanisms. First, microvascular obstruction induces distal ischemia [27] and disrupts local autoregulatory capacity [41], both directly contributing to neuronal damage. Second, thrombus components release neurotoxic mediators such as glutamate that further exacerbate neuronal injury [38]. In parallel, microvascular TI induces microglial migration and polarization toward a pro-inflammatory (M1) phenotype [42, 43]. All the TI phenomenon described above create an inflammatory milieu that propagates neuronal death, notably through apoptotic pathways [44].

However, it has been said that neuronal injury confined to these regions is probably insufficient to fully account for the neurocognitive deficits observed in rats, suggesting a more diffuse pattern of damage [39]. Such injury may involve a broader cortical distribution [45] or reflect more subtle alterations. For instance, a reduction in synapse density within the dendritic layer of the CA1 region has been reported in mice following experimental SAH [12].

### Neutrophils and NETosis

a.Preclinical evidence of neutrophil-driven thrombo-inflammation after aSAH.

During an experimental SAH, neutrophils [21] accumulate within microvessels [46], rapidly reaching the meninges and perivascular spaces [47], and subsequently infiltrating the brain parenchyma [48]. Neutrophils issued from the microcirculation enter perivascular spaces and then cerebral parenchyma via diapedesis through gaps formed by blood-brain barrier (BBB) disruption [48], a pattern reminiscent of observations reported in autopsy series of acute ischemic stroke (AIS) [49]. According to preclinical models of aSAH, BBB function, normally maintained by adjacent endothelial cells bound by thigh junction, is rapidly disrupted after aSAH [50], allowing immune cells infiltration [51]. Neutrophils likely play a direct role in BBB disruption, as inhibition of Toll-like receptor (TLR) 4, a key mediator of neutrophil NETosis, has been shown to attenuate BBB injury following experimental aSAH [52].

Recently, animal models have suggested that, in addition, a subset of neutrophils may be produced locally within the skull bone marrow before trafficking to the perivascular spaces and cerebrospinal fluid (CSF) via skull meningeal channels [53]. Notably, these neutrophils appear to exhibit a lower activation threshold for NETosis following aSAH [21].

In preclinical setting, adhesion of neutrophils to the microvascular endothelium [54] is promoted by increased expression of endothelial P-selectin [55], integrins [46], chemokines [56], and hemoglobin-degradation products [57] and occurs within 10 min after aSAH [58] before peaking on day 4 [59].

In the microvascular compartment, neutrophils contribute to microcirculatory occlusions [60], which, in animal models, leads to hypoperfusion in cortical territories [61]. As a surrogate marker of their detrimental activity, reducing neutrophil activation decreases early microvascular injury after aSAH in rats [54].

In preclinical models, neutrophil adhesion to the endothelium already promotes microglial polarization toward a pro-inflammatory (M1) phenotype and appears to drive microglial accumulation within the same brain regions [42].

In the microcirculation, CSF and parenchyma, neutrophils release pro-inflammatory mediators, myeloperoxidase (MPO), neutrophil elastase, metalloproteinase-9 (MMP-9), ROS, and NETs, responsible, beyond microthrombosis, for BBB disruption [54] and direct parenchymal injury [62]. Neutrophil activation is therefore associated with poorer neurological outcomes, especially cognitive impairment: in mice, neutrophil depletion improves memory, in part by restoring hippocampal N-methyl-D-aspartate receptor (NMDAR) function [63]. On the contrary, dysfunction of these receptors contributes to neuronal apoptosis after SAH [64] and to late long-term potentiation (LTP) deficits between 3 and 6 days after aSAH [63], whose role in memory consolidation is well known [65].

b. NETosis after SAH.

NETosis is defined as an active process of extrusion of decondensed chromatin fibers decorated with citrullinated histones and stabilizing proteins (i.e. chlorinated polyamines) into the interstitial and endovascular space by neutrophils along with other enzymes such as elastase, cathepsin G or MPO [66, 67].

**Fig. 1:**
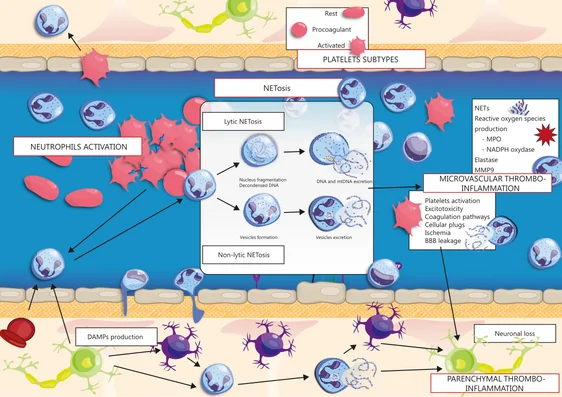
An overview of neutrophil extracellular traps formation in the microvessels and brain parenchyma. In preclinical models, neutrophils and platelets adhere to the activated endothelium within minutes after SAH, because of the adhesion molecules overexpressed by the activated endothelium or chemokines produced by platelets or brain cells. Thereafter, they may stay in the microvessels, organizing homotypic or heterotypic cells aggregates, or enter the brain through lesions -they are partly responsible for- in the blood-brain barrier. In the brain and the microvessels, neutrophils activate in response to the damage associated molecular patterns (DAMPs) excreted by dying neurons or cytokines produced activated platelets and microglia. Upon activation, neutrophils excrete the so-called neutrophil extracellular traps (NETs), composed of decondensed deoxyribonucleic acid (DNA) decorated with histones and pro-inflammatory enzymes, by two distinct mechanisms. The lytic NETosis involves nucleus fragmentation and cell necrosis, while the non-lytic NETosis necessitates the formation and the excretion avec vesicles containing the NETs. When they enter the extracellular space, NETs activate primary hemostasis (including platelets) and coagulation pathway as well as microglia (promoting type 1), leading to neuronal cell death, secondarily responsible for delayed cerebral ischemia and cognitive impairment. Abbreviations: BBB: blood-brain barrier, DAMPs: damage associated molecular patterns, NADPH: (nicotinamide adenine dinucleotide phosphate, NETs: neutrophil extracellular traps, MMP: metalloproteinase, MPO: myeloperoxidase. Created in Adobe Illustrator

Two distinct pathways, which differ from necrosis and apoptosis, may result in NETosis : the classical or lytic pathway, resulting in the death of the originating cell, and the vital pathway, where DNA is encapsulated in vesicles secondary expelled in the extra-cellular space [68]. Interestingly, mitochondrial DNA sometimes secreted along with DNA in the in vivo pathway [69] may also play a significant role by acting as a Damage-Associated Molecular Patterns (DAMP), creating an amplifying loop [70]. Both pathways are presented in Fig. 1 and may be associated under certain conditions [71].

Initially described as a host defense mechanism against bacteria following neutrophil stimulation by pathogen-associated molecular patterns (PAMPs) [72], these structures are now recognized as key mediators of sterile inflammation such as AIS.

Within all compartments, neutrophils are attracted and activated by a wide range of triggering signals. Among them, the more relevant are likely to be DAMPs released passively and actively by injured cells, hemoglobin-degradation products, and cells activated within microvessels or the brain parenchyma. Massive DAMPs release from injured cells is indeed a major activation signal. Among these, high-mobility group box 1 (HMGB1), produced locally [73] and immediately [74] by cells affected during early brain injury (EBI, during day 0 to 3 after SAH) [75] whose peripheral blood concentration is increased after SAH and correlates with DCI [76], potently stimulates neutrophil via activating receptors. Among them, the TLR superfamily, and notably the TLR4 [77] seems to be of paramount importance in NETosis triggering [78].

TLR4 is unique among pattern recognition receptors in its ability to activate both the myeloid differentiation primary response protein 88 (MyD88)–dependent and Toll/interleukin-1 receptor domain–containing adaptor-inducing interferon-β (TRIF - during the later phase of aSAH) signaling pathways and subsequent inflammatory reaction [79].

Heme present in subarachnoid clot degradation products (i.e. oxyhemoglobin [80, p. 4] and methemoglobin [81]) directly stimulates neutrophils TLR4 [80] signaling and activates the MyD88-dependent pathway during the early phase of SAH, which appears to be essential for NETs secretion [82]. MyD88 signaling induces nuclear factor-κB (NF-κB), which seems to be particularly active between days 3 and 5 after experimental aSAH in rabbits [83], as well as mitogen-activated protein kinase (MAPK) pathways, leading to apoptosis, pro-inflammatory gene expression [57], and NETosis [84]. Supporting the clinical relevance of this pathway during the early days of post-aSAH TI, pharmacological inhibition of TLR4 signaling reduced EBI in an experimental rat model of aSAH [85].

In parallel with TLR4 signaling, DAMPs also activate nicotinamide adenine dinucleotide phosphate (NADPH) oxidase [86], whose activation is required for NETs formation [87]. Erythrolysis degradation products also stimulate reactive oxygen species (ROS) [88] production, thus contributing to oxidative tissue damage and nitric oxide depletion (NO), leading to micro- and macro-vasospasm after SAH [89]. ROS themselves serve as potent triggering signals for NETs formation after hemorrhage [90].

Finally, peptidylarginine deiminase 4 (PAD4), which is essential for NETosis through histone citrullination [87], promotes chromatin decondensation and facilitates the extrusion of chromosomal deoxyribonucleic acid (DNA). PAD4 expression is increased in neutrophils after SAH [91], possibly in response to elevated intracellular calcium levels [92] secondary to NADPH oxidase–regulated ROS generation and subsequent Ca²⁺ influx [93].

**Fig. 2:**
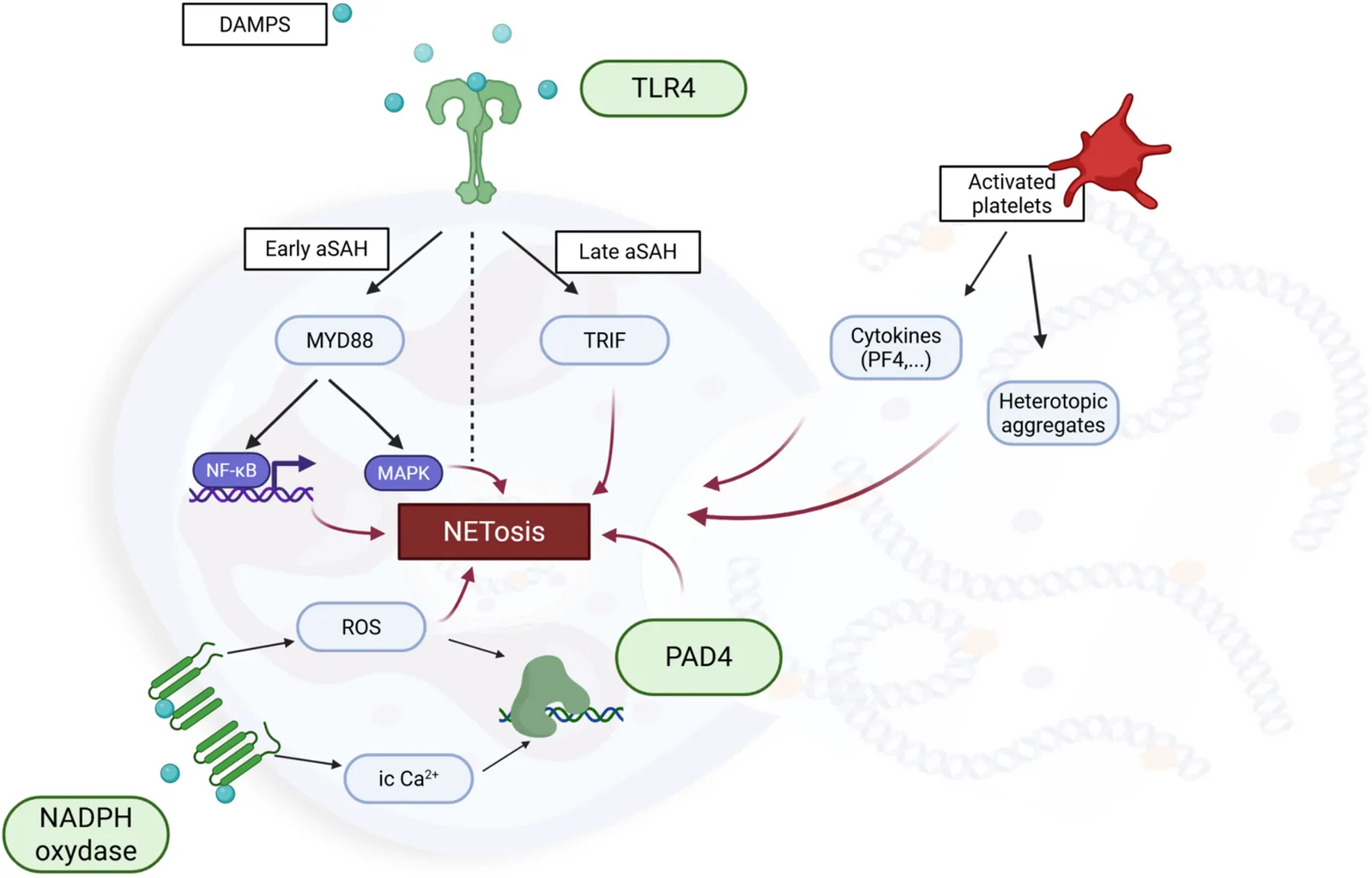
An overview of intracellular pathways involved in neutrophil extracellular traps formation. Damage-associated molecular patterns (DAMPs) released by cells (particularly distressed neurons) activate toll-like receptor 4 (TLR4), which is unique in that it triggers two intracellular thrombo-inflammatory pathways: MyD88 (predominantly within the first 5 days after SAH) and TRIF (activated later). Both pathways contribute to the induction of NETosis, with MyD88 acting via activation of NF-κB and MAPK signaling. DAMPs also stimulate NADPH oxidases, whose activation leads to reactive oxygen species (ROS) generation and increased intracellular calcium levels. These processes are directly and indirectly—via PAD4, which mediates histone citrullination and is essential for NETosis—involved in NETosis initiation. Activated platelets at the site of thrombosis also promote NETosis through aggregate formation and the secretion of pro-inflammatory cytokines, including PAD4. Abbreviations : BBB: blood-brain barrier, DAMPs: damage associated molecular patterns, NADPH: (nicotinamide adenine dinucleotide phosphate, NETs: neutrophil extracellular traps, MMP : metalloproteinase, MPO: myeloperoxidase, TLR: toll-like receptor. Created in Adode Illustrator and BioRender. Oury, C. (2026) https://BioRender.com/40vbtws

Platelets, which aggregate in the microvasculature immediately after SAH [94] in animal models and whose binding to neutrophils is associated with vasospasm [95], are regarded as potent activators of neutrophils [96] and NETosis [97]^,^ [98] (Fig. 1). Neutrophil activation by platelets occurs through direct binding to neutrophils with formation of heterotypic aggregates [99], enhanced after aSAH [58] and correlating with NETs formation [100] or through secretion of platelet-derived pro-inflammatory cytokines [97]. Among cytokines, Platelet Factor 4 (PF4, a potent neutrophil activator in neurovascular diseases [101]) concentration in peripheral blood has been correlated with perfusion impairment after aSAH [102]. Activating pathways involved in NETosis are illustrated in Fig. 2.

While NETosis has previously been implicated in microcirculation-related diseases (such as lupus, infectious diseases or AIS) [103–105] it has more recently been associated with DCI [21]. In murine experimental models of SAH, NETs are produced between 24 and 48 h post-SAH within microvascular spaces - where they are associated with microthrombosis [106] - and in perivascular spaces [107]. Their concentration then rises in both hemispheres of the brain until day 14 [108], paralleling the DCI window and the peak of neuronal death [40]. In mice, intravascular NETs correlate with microthrombi and with DCI [21], whereas perivascular NETs drive microvasospasm [107].

In humans, NETs concentration increases in CSF and blood after SAH [108] and correlates with aSAH severity [109]. In the recent paper of Schneider et al., their concentration rose until day 9 and then trended back down on day 10 [110]. In somewhat contradictory results, the occurrence of DCI relates either positively [110] or negatively [111] to the concentration of NETs, measured in peripheral blood as MPO-DNA complexes in the early phase of DCI (day 4 to 5 after SAH). The explanation proposed by the authors of the latter study is that NETs may have been consumed within thrombi, thereby reducing their measurable circulating levels. However, it should be noted that the method used to quantify NETs in this study is generally considered imprecise and may not accurately reflect the true burden of NET formation [112].

By contrast, most correlation studies in humans have relied on citrullinated histone H3 (CitH3). Elevated CitH3 levels have been associated with aSAH severity when measured within the first 24 h after ictus [109], and with the subsequent development of DCI when assessed at 2, 7, and 10 days after aSAH [21, 110]. Of note, Sajjad et al. found no association between CitH3 levels and angiographic vasospasm of the large cerebral arteries [113]. This observation is not necessarily inconsistent with the currently proposed role of NETs, which are thought to exert their deleterious effects predominantly at the microcirculatory level rather than through large-vessel vasospasm.

The involvement of NETs in DCI aligns with their strong pro-coagulant and pro-inflammatory potential. Positioned at the core of TI networks, NETs potentiate multiple pathways simultaneously, spanning platelet activation, coagulation cascades, complement engagement, and broader immune-cell activation. First, they provide a scaffold for platelets and immune cells adhesion and for local concentration of coagulation factors [114, 115]. Furthermore, they directly promote coagulation by binding circulating von Willebrand factor (vWF) and fibrinogen [115], by providing a procoagulant cofactor template for the factors XII and XI-induced contact activation and amplification of blood coagulation [116]. Furthermore, they are also decorated with tissue factor under certain conditions [117] and finally, they strongly stimulate platelet TLR4, enhancing thrombin generation [118]. Activated platelets further amplify microthrombosis [115], microvasospasm [119] and directly promote NETs production [72], creating a potential feed-forward loop. Beyond their role in TI, NETs may directly stimulate microglia. As noted above, M1-microglia stimulation contributes to neuronal injury via multiple mechanisms and, thereby, to cognitive deficits. One putative mechanism is binding of NETs components to microglial TLR4 receptors [120], whose expression increases after SAH [121] and whose activation is strongly pro-inflammatory [122], essential for neuronal injury [123] and for cognitive impairment in animal models of aSAH [124], yet another amplification loop in which NETs may play a central role.

Providing indirect evidence for a role of NETs in microglial polarization, intraperitoneal (50 µg) followed by intravenous (IV, 10 µg) administration of DNase I 3 h after an experimentally induced SAH in mice significantly reduced the level of pro-inflammatory subtype transition of microglia (marked with CD16/32, IL1β) and neuronal injury [125], although the precise spatial distribution of neuronal lesions was not reported. Interestingly, this reduction in microglial activation is accompanied by a significant decrease in ex vivo brain edema and a significant improvement in neurobehavioral tests (i.e. Garcia test).

Furthermore, reinforcing the concept of co-dependence between microcirculatory injury and microglial pro-inflammatory activation, microglial surface area—a proxy for microglial activation—is up to 40% greater (estimated marginal means [95% CI]; 6.1 [5.4–6.9] vs. 4.3 [3.6–5.0], *p* < 0.001) around thrombosed microvessels in patients, at a mean delay of 1 day [0.3–9] after aSAH [43], in an autopsy series comparing 11 aSAH and 10 control patients.

Finally, NETs have recently been involved in the development of a subacute hydrocephalus in a preclinical model of SAH. In this model of kaolin-induced hydrocephalus in rat, the intrathecal application of DNase I (3500UI during 3 days after hydrocephalus induction) prevented ventricular enlargement evaluated on T2-weighted MRI images 28 days after hydrocephalus induction [126]. The proposed mechanism is the reduction of the proliferation and differentiation of meningeal fibroblasts induced by NETs, otherwise contributing to aggravated subarachnoid fibrosis.

c. Systemic effects of NETs.

Despite their role in antibacterial defense, there is currently no demonstration that targeting NETs increases infection risk. On the contrary, it has been suggested that NETs secretion may limit immunoglobulin production by lymphoid organs, possibly by inducing B cell loss in Peyer’s patches via activation of apoptosis pathways [127]. Even in the brain, NETs may worsen infections: their concentration correlates with severity of pneumococcal meningitis in patients, and DNase I reduces bacterial load in an animal model of meningitis [128], possibly by improving bacterial clearance.

Conversely, some animal data suggest a link between circulating NETs and neurogenic pulmonary edema (NPE) [109], associated with an increased 1-year mortality after aSAH [10] while DNase administration [129] as well as P2 × 7R inhibition [130] (involved, among other roles, in NETs formation [131]) and melatonin administration [132] reduce NPE after experimental SAH in rats. In the latter study, MPO and MMP9, often used as proxy for neutrophil activation level, were reduced in the lungs of the treatment group. Furthermore, peripheral neutrophil count (but not NETs directly) is also strongly correlated with left ventricular dysfunction after aSAH [133].

In a nutshell, neutrophils, activated by both local and systemic signals, therefore mediate vascular, perivascular, and parenchymal injury that is detectable in humans. Animal models further indicate that this injury contributes to parenchymal, and specifically neuronal damage during the subacute phase of aSAH. Among neutrophil-derived effectors, NETs, composed of extracellular DNA networks, have been repeatedly correlated with clinical outcomes in patients with aSAH, while their therapeutic targeting in preclinical models attenuates the cognitive deficits associated with aSAH.

### Effects of NETs Inhibition

Targeting NETs can be achieved through several strategies: reducing neutrophil activation (for instance by limiting DAMP–neutrophil interactions [134]), inhibiting NETs formation pathways (e.g., by inhibiting enzymes required for NETs production such as PAD4, NADPH oxidase, or MPO), or degrading extracellular DNA networks once released, through DNase administration [135].

Given the very large number of preclinical studies aimed at inhibiting -at least in part- the effects of neutrophils activation after aSAH, we chose to prioritize animal studies focusing on pathophysiological pathways that focus mainly on neutrophils and can already be targeted in humans, either with novel agents or through drug repurposing.

We thus examine, along the NETosis pathway, an example of a receptor stimulated by DAMPs (TLR 4), an enzyme required for NETs release (MPO), and an enzyme co-released with NETs by neutrophils (MMP-9), as well as the lysis of NETs themselves by DNase—all of which may represent targets for potential trials using existing drugs.

It should be noted that in these studies, the primary outcome rarely involves direct assessment of DCI, but rather relies on proxies—typically imaging or biological biomarkers or functional clinical assessments—that can be linked to the occurrence of DCI.

#### NETs Secretion Inhibition

TLR 4 inhibition, involved in NETosis in response to DAMPs [78, 81], has been evaluated in a positive phase 2 study in AIS [136]. In experimental aSAH models in mice, intraventricular administration of a TLR4 inhibitor 30 min after the SAH reduced BBB permeability in one study [52], and decreased cerebral edema while improving neurocognitive function in another [137]. Although NETosis was not directly investigated in either study, the inhibitors used may have exerted part of their effects through modulation of NET formation, potentially via the JNK pathway [138] in the first study and the MAPK and NF-κB pathways in the second.

MPO, required for NETosis [139] and endowed with intrinsic TI activity [140], has already been investigated in several diseases [141], including neurodegenerative conditions [142]. In this context, intraperitoneal administration of an MPO inhibitor for 4 days after experimental aSAH in mice appears to inhibit intrathecal neutrophils translocation—remaining confined within meningeal vessels—and improves spatial memory [143]. Earlier work from the same group suggested that the effects of neutrophils and MPO may be exerted indirectly through astrocyte dysfunction, with secondary neuronal injury [47].

MMP-9, co-released with NETs by activated neutrophils, contributes to BBB degradation. Its levels in CSF or blood following aSAH have been shown to predict the occurrence of DCI, vasospasm, and clinical outcome in several patient series [144–146].

Inhibition of MMP9 via minocycline administration in preclinical aSAH models in mice resulted in reduced vasospasm and improved neurobehavioral outcomes [147]. It is worth noting that minocycline is not only an inhibitor of MMP-9 but also of PAD4 [148], a key protein for NETosis [149].

#### NETs Degradation

As demonstrated in thrombus lysis experiments, where DNase effectively disrupts thrombi while leaving associated proteins and enzymes intact [103], the structural integrity of NETs appears to be fundamental to their deleterious effects. Consequently, degradation of this structure comes across as an interesting and somewhat critical therapeutic mechanism. As previously mentioned, NETs dismantling requires the action of a specific extracellular hydrolase—DNase—which hydrolyzes DNA phosphodiester bonds, followed by intracellular degradation by macrophages and monocytes [150]^,^ [151].

Two endogenous DNase families (DNase I and DNase II) exist; however, only DNase I functions in the extracellular compartment. The DNase I family comprises four members, DNase I, DNase1L1, DNase1L2, and DNase1L3, among which DNase I and DNase1L3 have been identified as the principal enzymes responsible for NETs degradation [150].

In the specific context of aSAH, several converging lines of evidence support the efficacy of DNase in reducing NETs burden and, beyond this, improving clinical outcomes. First, in humans, a reduction in endogenous DNase activity between days 0 and 4 after aSAH is associated with an increased risk of DCI [152], notably during the peaking of DCI risk. In mice, IV or intraperitoneal DNase administration after experimental SAH reduces NETs concentration and improves functional outcomes by directly enhancing cerebral perfusion through reduced microthrombosis [108, 109]. Within the parenchyma, NETs degradation also attenuates neuronal apoptosis [109].

In a striking study by Hao and colleagues, DNase reduced microthrombosis after experimental SAH in mice, leading to attenuation of brain edema, BBB disruption, neuronal injury, perfusion deficits, CSF flow dysfunction, and ultimately neurological impairment [153]. In another murine model, NETs targeting with DNase also reduced microvasospasm [107]. Importantly, DNase administration reverses the M1-microglial activation described above in mice [125].

Zeineddine et al. reported contrasting findings in another murine study [21]. Although DNase administration effectively reduced circulating NET levels, it failed to improve cognitive outcomes. While the dose used was comparable to that employed in previous studies, the route of administration differed, being exclusively intraperitoneal rather than partially intravenous or intracisternal. In addition, the second dose was administered later than in earlier reports. These methodological differences may partly account for the lack of cognitive benefit observed despite successful NET reduction.

Beyond the brain, DNase may also mitigate systemic complications of aSAH. In particular, a mice model by Wu and colleagues suggested that DNase reduces NPE after experimental SAH [129], indicating that its benefits may extend beyond cerebral and cognitive outcomes.

Taken together, these data support the hypothesis of a potential efficacy of DNase in reducing TI after aSAH. Overall, there is growing consensus coming from preclinical and clinical data that the time has come for a randomized controlled trial targeting NETs to prevent or treat DCI and cognitive impairment following aSAH [154].

### Clinical Trials

#### Thrombo-inflammation After aSAH

The research flowchart, along with all past and ongoing RCTs classified by outcome and target pathway, is provided as Supplemental Fig. 6.

To our knowledge, after our large review of trials dedicated to aSAH, the number of RCTs specifically targeting neutrophils in the acute phase of aSAH remains scarce, underscoring a significant contrast with the larger body of preclinical studies.

#### NETs Secretion Inhibition

Among currently ongoing clinical investigations, the compound most directly targeting NETs release appears to be SXN-CVS (NCT06138353), which inhibits the TLR4/NF-κB signaling pathway. This phase 2 study, planned to enroll 50 patients, has not yet reported results (initial recruitment period planned for 2024–2025).

Beyond that, among notable trials partially targeting TI with published results, a monocenter phase 2 trial in 48 patients, showed that randomized, placebo-controlled dapsone administration significantly reduced DCI (26.9% vs. 63.6%, *p* = 0.011) and emergent endovascular treatment of vasospasm (15.4% vs. 45.5%, *p* = 0.029) [155]. Dapsone exerts part of its effect by inhibiting MPO [156], which is required for NETs formation [139]. The MASH study, targeting MMP-9, a predominantly neutrophilic secretion, showed a decrease in BBB permeability quantitated by contrast signal intensity ratios (0.23 vs. 0.28, *p* < 0.01) [157]. Although the effect is less direct, a randomized, placebo-controlled erythropoietin administration reduced cerebral infarcts as a secondary endpoint (32.5% vs. 2.5%, *p* < 0.001) [158], potentially by acting on microglia polarization [159].

#### NETs Degradation

Several clinical studies conducted in the 1950s evaluated high-dose IV bovine DNase in infectious diseases (meningitis, abcesses) without reporting major complications [160–163], yet no IV formulation of DNase has since reached the clinical market. The given dose in these studies is difficult to interpret, as it is expressed in units derived from a viscometric assay, reflecting enzymatic activity rather than a standardized mass. Dornase alfa (Pulmozyme^®^, Roche), is a recombinant human DNase I (rhDNase), glycosylated and phosphorylated, authorized in many countries to improve pulmonary function by reducing bronchial congestion in patients with cystic fibrosis. In this indication, administration is by inhalation via nebulization. A 1999 study assessed IV Pulmozyme^®^ in patients with lupus, including safety and pharmacokinetics, and reported no serious adverse events [104]. In this study, rhDNase was well tolerated without significant adverse events following administration of 2 series of 5 consecutive days with a 2-day washout in between, and treatment was not associated with the development of neutralizing antibodies to rhDNase. Serum rhDNase concentrations (80-100ng/mL) capable of hydrolytic activity of rhDNase were achieved up to 24 h following IV injections of 125microgr/kg.

RESET (NCT06723717) is currently the only reported RCT aiming to improve medium-term functional, especially cognitive, outcomes in patients with aSAH by targeting microvascular and parenchymal NETosis through IV rhDNase (Dornase alfa, Pulmozyme^®^, Roche) daily administration. The primary objective of this phase 2b RCT is to evaluate the efficacy of daily IV infusion of dornase alfa (125 µg/kg) up to 14 days post-aSAH on the occurrence of an excellent functional outcome at 6 months, defined as a score of 0 or 1 on the modified Rankin Scale (mRS) at 6 months. Patients may be included up to 48 h after the onset of symptoms related to aSAH. The inclusions are ongoing, and the results are expected in 2028.

#### Other Trials

Outside the scope of our review but intersecting the pathways cited above, it is worth noting the following RCTs : CIAO\@SAH (NCT06359782), a phase 2 study of a C1 esterase inhibitor (C1-INH) expected to complete in 2027; FINISHER (NCT04566991), giving dexamethasone (recruitment completion was expected in 2025); BLISS (NCT06439615), inhibiting the Janus kinase 2/signal transducer and activator of transcription 3 (JAK2/STAT3) pathway – involved in M1-type microglia promotion and inducible nitric oxide synthase, interleukin-1β, and tumor necrosis factor-α after aSAH [164]- to prevent lung injury after aSAH (completion 2027) or THRIVE (NCT06863480), in which an interleukin-6 receptor (IL6R) blocker is given to reduce DCI (recruitment ends 2028).

To date, safety concerns about TI reduction after aSAH are limited. None of the halted trials were stopped for safety reasons or hemorrhagic excess (see Supplemental Fig. 3). Even administration of antiplatelet therapy in acute SAH (iSPASM, NCT03691727) has been shown to be safe, without excess of significant hemorrhage [165].

### Perspectives

As the first clinical trial specifically aiming to degrade NETs in the acute phase of aSAH has only recently been initiated, clinical data remains limited, precluding definitive conclusions at this stage. However, as observed in other neurovascular disorders, future therapeutic strategies are likely to rely on a combination of targets and complementary mechanisms of action.

In this regard, the relatively recent demonstration of the efficacy of lumbar CSF drainage [166] and efficacy of intracisternal DNase administration in animal models [107], invites consideration of adjunctive therapeutic delivery via this route, in addition to the IV route. Such an approach could allow targeting intrathecal inflammation in parallel with intra- and perivascular inflammatory processes.

Moreover, the combination of multiple DNase isoforms active against extracellular DNA, such as DNase I and DNase1L3, has shown enhanced NETs-degrading activity in preclinical models [167]. Their combined use therefore warrants evaluation in a clinical setting.

Finally, longitudinal studies have demonstrated temporal fluctuations in the relative intrathecal concentrations of leukocyte subtypes, with neutrophils [168] and their activation markers increasing before day 4 [56], preceding a secondary rise in lymphocyte concentrations occurring between days 5 and 9 [169]. However, most longitudinal studies have focused on single cell lineages, with limited sampling timepoints and minimal clinical correlation. These limitations are addressed by integrative cohorts such as RADICAL (NCT04971564) and TARGETS (NCT07354321), in which biological samples are collected at multiple timepoints during both the acute and chronic phases. Advanced analytical approaches, including spectral cytometry [170], will enable identification of leukocytes (and platelets) subpopulations specifically associated with clinical outcomes and thus representing priority therapeutic targets. In this context, the emergence of nanobodies, allowing highly specific and coordinated targeting of multiple markers of interest [171], appears particularly promising, both from a diagnostic and therapeutic point of view.

This review would be incomplete without addressing the numerous questions currently raised by translational research on post-aSAH TI. Indeed, several articles have been subject to an expression of concern [172] or retraction [173]. In some cases, the responses are still awaited, and the papers remain accessible. This situation — including the latency period during which the validity of the data has not yet been formally challenged — has itself been the subject of a recent publication [174]. As research on post-aSAH inflammation increasingly moves to the bedside, the acquisition of reliable and reproducible preclinical data has become a critical challenge.

## Conclusion

The growing body of ongoing trials targeting complementary TI pathways highlights a paradigm shift: moving beyond vasospasm-centered interventions toward integrated modulation of neuroinflammation and microvascular thrombosis.

Among the identified TI phenomenon, NETosis emerges as a pivotal processes bridging vascular injury, coagulation, and neuroinflammation after aSAH. By promoting microvascular obstruction, BBB disruption, and microglial activation, NETs may orchestrate both early and delayed secondary brain injury, contributing to DCI and cognitive impairment.

Evidence from preclinical models consistently suggests that NETs degradation using DNase mitigates microthrombosis, reduces neuronal apoptosis, and improves both cerebral perfusion and neurological recovery, yet without apparent immunosuppressive risk. These findings converge with the rationale of the RESET trial (NCT06723717), aiming to translate DNase therapy into the clinical management of aSAH.

This trial, among others, may help to define the optimal timing, dosing, and combination of anti-NETs and anti-neutrophil activation strategies to improve both survival and long-term neurocognitive outcomes after SAH. In this regard, the rapid development of neutrophil activation after aSAH could represent another paradigm shift, by redefining the degree of urgency required for the biological management of inflammation in this setting, bringing it closer, for instance, to that of AIS.

## Supplementary Information

Below is the link to the electronic supplementary material.


Supplementary Material 1


## Data Availability

No datasets were generated or analysed during the current study.

## Abbreviations

AIS: acute ischemic stroke
aSAH: aneurysmal subarachnoid hemorrhage
BBB: blood–brain barrier
CSF: cerebrospinal fluid
DAMPS: damage-associated molecular patterns
DCI: delayed cerebral ischemia
DNA: deoxyribonucleic acid
(rh)DNase: (recombinant human) deoxyribonuclease
DSA: digital subtraction angiography
EBI: early brain injury
GCS: Glasgow coma scale
HMGB1: high-mobility group box 1
JAK2/STAT3: Janus kinase 2/signal transducer and activator of transcription 3
LTP: long-term potentiation
MMP: metalloproteinase
MPO: myeloperoxidase
MRI: magnetic resonance imaging
NETs: neutrophil extracellular traps
NF-κB: nuclear factor-kappa B
NMDAR: N-methyl-D-aspartate receptor
NPE: neurogenic pulmonary edema
RCT: randomized clinical trial
ROS: reactive oxygen species
RSS: really simple syndication
SANRA: scale for the assessment of narrative review articles
tGCI: transient global cerebral ischemia
TI: thrombo-inflammation
TLR: Toll-like receptor
