# Supplementary material for: Impact of Neutrophil Extracellular Traps on Clinical Outcome after Subarachnoid Hemorrhage: A Translational Narrative Review
Source: Transl Stroke Res. 2026 Jun 30;17(4):73. doi: 10.1007/s12975-026-01461-6 (PMC13319143; doi:10.1007/s12975-026-01461-6)
Supplement: Supplementary file 1 — Supplementary Material 1 [file 12975_2026_1461_MOESM1_ESM.docx]

**Impact of Neutrophils and Neutrophil Extracellular Traps on Clinical Outcome after Subarachnoïd Hemorrhage : A Translational Narrative Review**

**Supplemental Data**

***Supplemental Table 1. Literature search: keywords***

THROMBO-INFLAMMATION AND SUBARACHNOID HEMORRHAGE

| TERMS | RESULTS (OVERALL) | DATE |
| --- | --- | --- |
| ("Subarachnoid Hemorrhage"[Mesh] OR "subarachnoid hemorrhage"[tiab] OR "aneurysmal subarachnoid hemorrhage"[tiab] OR "aSAH"[tiab])  AND  ("Thrombosis"[Mesh] OR "Inflammation"[Mesh] OR "Blood Coagulation"[Mesh] OR "Neuroinflammation"[Mesh] OR "Platelet Activation"[Mesh] OR "Neutrophil Extracellular Traps"[Mesh]  OR thromboinflammation[tiab] OR "thrombo-inflammation"[tiab] OR thrombosis[tiab] OR inflammation[tiab] OR "blood coagulation"[tiab] OR neuroinflammation[tiab] OR "platelet activation"[tiab] OR "neutrophil extracellular traps"[tiab] OR NETs[tiab])  AND  ("2014/07/01"[Date - Publication] : "3000"[Date - Publication]) | 1797 | 08/12/2025* |

NETOSIS AND SUBARACHNOID HEMORRHAGIE

| TERMS | RESULTS (OVERALL) | DATE |
| --- | --- | --- |
| "Subarachnoid Hemorrhage"[Mesh] OR "subarachnoid hemorrhage"[tiab] OR "aneurysmal subarachnoid hemorrhage"[tiab] OR "aSAH"[tiab]) AND ("Neutrophil Extracellular Traps"[Mesh] OR "neutrophil extracellular traps"[tiab] OR "NETs"[tiab] OR "NETosis"[tiab] OR "NET-osis"[tiab]) AND ("2014/07/01"[Date - Publication] : "3000"[Date - Publication]) | 24 | 08/02/2026* |

*An RSS feed was systematically followed during the preparation phase in order to capture and integrate the most recent relevant articles into the review

1) Inclusion criteria

1. Population or model: Studies involving animal models of experimental subarachnoid hemorrhage or human subjects with aneurysmal subarachnoid hemorrhage.

2. Pathophysiological focus: Studies investigating neutrophil activation and NETosis.

3. Study type: Preclinical studies, observational clinical studies, and randomized controlled trials.

4. Temporal relevance: Studies focusing on the acute or subacute phases of aSAH, particularly within the time window of delayed cerebral ischemia (up to day 21).

5. Outcomes: Studies reporting mechanistic insights, biological markers, imaging findings, or functional/clinical outcomes relevant to thrombo-inflammation after aSAH. As DCI is difficult to assess in animal models, we accepted biological, clinical, or imaging biomarkers that may be linked to DCI occurrence.

6. Translational relevance: Priority was given to studies investigating pathways that can be targeted in humans, either through novel agents or drug repurposing strategies.

7. Additional references: Selected references were included for clarification and educational purposes, including closely related thrombo-inflammatory conditions and pathways associated with NETosis when mechanistically relevant.

2) Exclusion criteria

1. Case reports.

2. Technical papers focusing on vasospasm treatment.

3. Articles addressing other forms of stroke or combining multiple types of brain injury.

4. Articles not published in English.

5. Papers published in non–peer-reviewed journals or predatory journals.

6. Meta-analyses and consensus statements were used for cross-checking references but were not directly cited in the text, except when illustrating phenomena peripheral to the main focus of the review.

3) Quality assessment

Quality assessment was based on two established tools for systematic reviews of animal studies: the SYRCLE risk of bias tool^1^, adapted from the Cochrane RoB tool, and the CAMARADES checklist^2^.

Given that many studies involved small sample sizes, emphasis was placed on methodological description, internal validity, and reproducibility; relevance of the primary outcome; allocation concealment when reported; transparency regarding study limitations; and publication in peer-reviewed journals. Studies published in predatory journals were excluded.

As randomized animal studies remain limited in this field, randomization was not considered mandatory but was regarded as a quality indicator when present. Finally, translational applicability—particularly the feasibility of targeting the identified pathways with existing therapies—was a key selection criterion, in line with the translational objective of this review.

**Supplemental Figure 1. Literature search : flowchart**


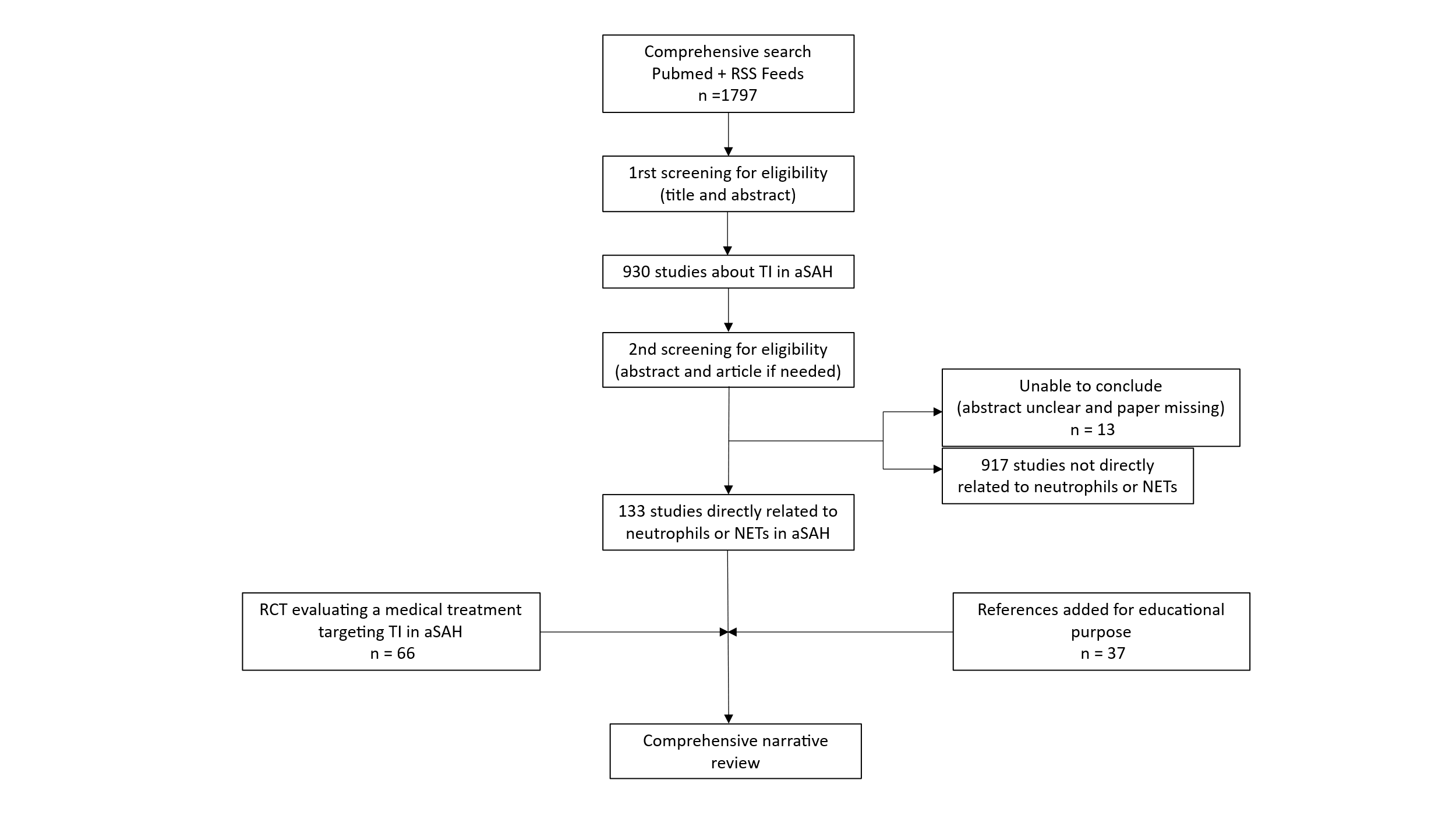

**Supplemental Figure 2. RCTs on TI after aSAH published on clinicaltrials.gov : flowchart**

**Supplemental Figure 3. RCTs on TI after aSAH published on clinicaltrials.gov : unpublished or halted RCTs**

**Supplemental Figure 4. RCTs on TI after aSAH published on clinicaltrials.gov : negative results**

**Supplemental Figure 5. RCTs on TI after aSAH published on clinicaltrials.gov : positive results**

**Supplemental Figure 6. RCTs on TI after aSAH published on clinicaltrials.gov: ongoing trials**

**References**

1. Hooijmans CR, Rovers MM, de Vries RB, Leenaars M, Ritskes-Hoitinga M, Langendam MW. SYRCLE’s risk of bias tool for animal studies. *BMC Med Res Methodol*. 2014;14:43. doi:10.1186/1471-2288-14-43

2. Macleod MR, O’Collins T, Howells DW, Donnan GA. Pooling of animal experimental data reveals influence of study design and publication bias. *Stroke*. 2004;35(5):1203-1208. doi:10.1161/01.STR.0000125719.25853.20
